# On the ontological assumptions of the medical model of psychiatry: philosophical considerations and pragmatic tasks

**DOI:** 10.1186/1747-5341-5-3

**Published:** 2010-01-28

**Authors:** Tejas Patil, James Giordano

**Affiliations:** 1 Keck School of Medicine, University of Southern California, 1975 Zonal Avenue, Los Angeles, California 90089, USA; 2 Center for Neurotechnology Studies, Potomac Institute for Policy Studies 901 N Stuart St, Suite 200, Arlington, VA 22203, USA; 3 Wellcome Centre for Neuroethics, University of Oxford, OX1, 1PT, Oxford, UK; 4 Uehiro Centre for Practical Philosophy, University of Oxford, OX1, 1PT, Oxford, UK

## Abstract

A common theme in the contemporary medical model of psychiatry is that pathophysiological processes are centrally involved in the explanation, evaluation, and treatment of mental illnesses. Implied in this perspective is that clinical descriptors of these pathophysiological processes are sufficient to distinguish underlying etiologies. Psychiatric classification requires differentiation between what counts as normality (i.e.- order), and what counts as abnormality (i.e.- disorder). The distinction(s) between normality and pathology entail assumptions that are often deeply presupposed, manifesting themselves in statements about what mental disorders *are*.

In this paper, we explicate that realism, naturalism, reductionism, and essentialism are core ontological assumptions of the medical model of psychiatry. We argue that while naturalism, realism, and reductionism can be reconciled with advances in contemporary neuroscience, essentialism - as defined to date - may be conceptually problematic, and we pose an eidetic construct of bio-psychosocial order and disorder based upon complex systems' dynamics. However we also caution against the overuse of any theory, and claim that practical distinctions are important to the establishment of clinical thresholds. We opine that as we move ahead toward both a new edition of the Diagnostic and Statistical Manual, and a proposed Decade of the Mind, the task at hand is to re-visit nosologic and ontologic assumptions pursuant to a re-formulation of diagnostic criteria and practice.

## Introduction

Psychiatry is uniquely problematic because debates over what mental disorders *are *have presented substantial challenges to medical praxis and ethics. In many ways, the question of what constitutes a mental disorder is related to uncertainties about the nature of mental experience, and the underlying relationship(s) of body, brain and mind. Traditionally, medicine has been successful in establishing etiology of diseases and disorders, and developing focal therapies based upon such mechanistic conceptualizations. The acts of medicine (i.e.- diagnosis, therapeutics, and prognosis) depend upon the ability to distinguish between what is "normal" and what is pathologic, and the evolution and practice of psychiatry has attempted to adopt and utilize the medical model in this regard.

Yet, as neuroscience probes ever deeper into the workings of the brain, it becomes evident that the "mind" remains somewhat enigmatic, and thus, any attempt to link mental events to biology must confront what Chalmers has referred to as the "hard problem" of consciousness [1]. But given the continued ambiguity of the brain-mind relationship, unresolved questions remain of 1) how can, and perhaps should psychiatry proceed to formulate a viable system of characterizing mental normality and abnormality, and 2) how might such formulation affect the scope and tenor of psychiatric practice?

As several papers in this journal have shown, such questions are not esoteric or merely academic. Rather, in light of 1) ongoing progress in genetics and neuroscience; 2) development and tentative articulation of a forthcoming Decade of the Mind; and 3) proposed healthcare reforms that are based to a large extent upon diagnostic classifications, these questions reveal genuine challenges, and form the groundwork upon which a new diagnostic schema (if not *Diagnostic and Statistical Manual*) for, and definition of psychiatric profession and practice might be constructed.

## Discussion

### Problems in Psychiatric Diagnosis

Horwitz asserts that "because [diagnostic psychiatry] uses symptoms to classify disorders, it also categorizes an enormous diversity of human emotions, conduct, and relationships as distinct pathological entities" [2]. At first blush, such an approach seems logical because precise diagnostic classifications can presumably distinguish between particular disease states and offer reliable information about etiology, prognosis, and treatment. In the *The Myth of Mental Illness*, Szasz disputed psychiatry's claims of medical legitimacy. Szasz was concerned about the validity of psychiatric concepts, and his critique raised questions about the evaluative nature of the psychiatric enterprise. To Szasz, psychiatry utilized terms (such as delusions, compulsions, and obsessions) that lacked the descriptive objectivity of other domains of medicine. Szasz did not deny that neuroanatomical lesions could result in dysfunctional behaviors, however, such abnormality is, strictly speaking, a brain disease. Labeling various forms of behavior as pathological "...rests on a serious, albeit simple, error: ... mistaking or confusing what is real with what is imitation; literal meaning with metaphorical meaning; medicine with morals" [3]. If psychiatry lacked terms that could definitively individuate normality from pathology, how could psychiatrists issue seemingly objective diagnoses and prognoses while relying on a predominantly subjective (and elastic) epistemology?

This conceptual tension in psychiatry mirrors larger debates about objectivity and normativity in the philosophy of science. In *The Structure of Scientific Revolutions*, Thomas Kuhn argued that science does not operate within an Archimedean framework, but instead, is sensitive to the normative practices of social communities [4]. Scientists (and clinicians) undergo training and develop expertise within localized academic institutions. As a consequence, intellectual traditions tend to bind scientists and clinicians within a coherent community of practitioners. Kuhn noted that members of a particular academic community tend to hold similar constructs and values about what constitute a good theory, and these values were largely assumed, unquestioned, and maintained as valid within the group. For Kuhn at least, the collective nature of scientific theory-building suggested that communities' *values *matter in the content of scientific discourse and theorization (and, we might add, clinical practice).

Postmodern criticisms of science generally impugn this relativistic bend, and pose the question: If science evolves within a cultural frame (just like other ideologies), then in what sense is it immune from the normative practices of society [5]? The crucial issue is not whether the unique status of science (and by extension, clinical medicine) hinges on cultural biases, but whether its epistemology is better than other ideologies at obtaining knowledge about the natural world. All ideologies manifest hegemonic assumptions about the nature of reality and being. However, unlike other ideologies, science also values a self-correcting process through which increasingly refined and robust characterizations about the natural world can be made over time. If new observations become difficult to reconcile with standing hegemonic beliefs, then those initial assumptions are usually abandoned. Thus, scientific epistemology allows for large scale reorganization of ontological assumptions, or what Kuhn called "paradigm shifts" [4].

In applying this framework to the medical model of psychiatry, we see a reliance upon four main ontological assumptions. These are 1) *Realism*: the claim that mental properties (such as desires, beliefs, and thoughts) are real phenomena and not merely artifacts of socio-cultural norms; 2) *Naturalism*: the concept that disturbances in neural structures are causally implicated in the formation and persistence of mental disorders; 3)*Reductionism*: the view that at some level, disturbances in neural structures are necessary to account for mental disorders, and 4) *Essentialism*: the assertion that mental disorders have underlying 'essences" that allow distinction of one type from another.

Are each and all of these assumptions warranted and necessary in order to arrive at a valid concept of mental disorder? We assert that naturalism, realism, and reductionism are reconcilable with advances in contemporary neuroscience, but that essentialism has proven to be, and may still be somewhat more problematic, vis-a-vis the medical model of psychiatry, at least to date. Let us examine each of these assumptions in turn.

### Realism

The realist position asserts that terms used in scientific theories map onto actual properties in the external world, even if the relevant phenomena are not necessarily observable. So, for example, sodium-gated ion channels or serotonin receptors all do, in fact, exist. Their existence is not predicated upon our ability to perceive them through our senses. Another important aspect of realism is that properties referred to by scientific theories are independent of our linguistic practices or socio-cultural norms; hence, the amino acid glycine will always have a hydrogen atom as its functional group. This description holds true regardless of human circumstance.

Realism entails that a mental realm does not exist separately from the physical, and so an acceptance of realism necessitates a rejection of dualism. Simply, there is not an ontologically separate mental world, independent of its physical instantiation in the brain. The idea of an overriding mind, metaphysically independent of the brain, becomes untenable when we realize that lesions to various regions of the brain have profound consequences for subsequent subjective experience. How would the mental realm causally interact with an aphasic's brain, given the loss of linguistic capabilities due to an insult to the superior temporal gyrus or Broca's area? Similarly, how are we to account for the gradual loss of cognitive function in patients with Alzheimer's disease?

To experience disease is to *be *in a certain experiential state. To use a rather overplayed computational metaphor, to have such an experience requires that one have the requisite "hardware" (brain) and "software" (mind). A rejection of dualism would logically mean that all mental disorders are (in some way) biologically based. The tenet claims that every mental process, pathological or otherwise, arises in and from the brain [6]. It is important to note that nothing has been claimed about *how *neural structures causally produce mental states (*naturalism*), or whether mental states are best understood through their more basic, physical components (*reductionism*).

Realism has been a rather controversial assumption in the philosophy of psychiatry. An objection to the realist case is that there is no reason to claim that mental properties, such as beliefs, doubts, desires, and fears actually exist in the natural world. Moreover, as matter of fact, such mental properties do depend on the normative constraints of local communities. According to Cash, "...people's intentions, beliefs, thoughts and decisions are different in kind, not just in scale, from causal mechanisms in the brain. The nature of this 'difference in kind' can be revealed by considering the nature of the public criteria we use to ascribe intentional states to one another" [7]. The veridicality of intentional states often depends upon the requisite conditions; intentional states can *mean *or *be about *something. The property of *aboutness *cannot be mapped onto reality in any law-like way.

One can sidestep this criticism by noting that realism is best approached as an epistemological constraint. It is not the case that the tentative plausibility of a certain theoretical term commits us to finding its 'real world" equivalent. The validity of theoretical terms, that is, their ability to appropriately map onto real world properties, is completely contingent on the congruency of the associated theory with other established scientific principles. Critics of realism often conflate the object of scientific knowledge with the process of knowledge construction. Fundamentally, science is an interpretative process; it is something people *do*. Given that science is a project of collaboration, it is empirically impure, relying on built-in explanations that become embedded in the process of theory development. This does not mean that science is merely a by-product of cultural practices. Roy Bhaskar articulates the problem in this way:

"[M]en in their social activity produce knowledge which is a social product much like any other, which is no more independent of its production and the men who produce it than motor cars, armchairs and books... and which is no less subject to change than any other commodity. This is one side of 'knowledge'. The other is that knowledge is *'of' *things which are not produced by men at all: the specific gravity of mercury, the process of electrolysis, the mechanism of light propagation. None of these 'objects of knowledge' depend upon human activity. If men ceased to exist sound would continue to travel and heavy bodies fall to earth in exactly the same way, though *ex hypothesi *there would be no one to know it" [8].

Knowledge, in the form of theories and explanations, is interpretational and should be regarded as a changeable social product. This does not mean that the object of any such knowledge is always dependent upon socio-cultural constructions. Science describes entities of nature, but "proof" comes through our success in interpreting, interacting with, manipulating (and often, controlling) them.

### Naturalism

Naturalistic theories of mind generally assume that mental properties, such as thoughts or beliefs, are derived from neurobiological structures in a causally relevant way. In order to legitimize the naturalistic characterization of a mental disorder, the observed clinical expressions of behavior should have causal roots in biology. This is not to claim that all mental behavior should *only *be understood through biology, but rather that we - as dynamic organisms within complex environments - will undoubtedly be influenced by a variety of interacting variables, including biology.

A pressing question in naturalistic theories is how is it, exactly, that neurobiological disorders can be causally linked to certain behavioral outcomes? The steps implicated in the causal chains from the biochemical to the behavioral level(s) are vast and endless, and as Hume noted, we cannot "see" causation [9]. In science, we observe event regularities, and if such regularities occur with sufficient frequency, then we tentatively accept these observations as truly causal. Such observations are affirmed through the use of statistical theories, which provide a mathematical measure for the probability of an event occurring solely by chance.

While the development of statistical methods has refined the scientific process, the act of establishing causal relationships in the world long predates the development of statistics, or even mathematics. Such reasoning is possible because human beings have the capacity to reason inductively and infer logical relationships from data in, and obtained from the environment. Children as young as three years old can make appropriate judgments about novel stimuli and causally link processes they have only observed in operation [10].

These types of observations have prompted many philosophers (since Hume) to posit that causality can, at best, be understood as event regularities. We cannot determine by reasoning alone which of the observed (or potentially unobserved) effects actually cause the phenomena in question. To arrive at such conclusions, however, is to be led astray by words. As Ross states, "...to the extent that we have culturally universal intuitions about causation, this is a fact about our ethology and cognitive dispositions, rather than a fact about the general structure of the world" [11]. In other words, naturalistic intuitions are not evidence of their content.

### Reductionism

Over the last few decades, neuroscience has elucidated a biological basis for several mental disorders. These developments have fuelled the quest to explain mental properties by reducing them to an interaction of their putative substrates. Given that interactions of neurobiological structures are causally implicated in aberrant of behavior, a logical paradigm would grant underlying genetic and biochemical entities explanatory primacy. Subjective experience and cultural influences can play a role in psychiatric disorders, but the "true" explanatory locus would rest in pathological structures and functions.

Many of these overly reductionist tendencies can be assuaged by revisiting some of Dennett's work that attempts to clarify the relations and predictions of mentalistic behavior through the use of three levels of explanatory abstraction [12]. The first is the *Physical Stance*, in which behavior could be predicted, in principle, from physical laws governing the interactions of material components. The second is the *Design Stance*, which predicts behavior, not from an understanding of the physical constitution of the mind, but through an understanding of the mind's purpose, function, and design. The final level of abstraction is the *Intentional Stance*, which requires neither an understanding of the physical constitution of the mind nor any design principles, but instead predicts behavior by considering what moves a rational agent would make in a given circumstance.

The brain and its potential representations are a primary focus of neuroscience, and neuroscientific information sustains both an evolving philosophy of mind, and the profession and practice of psychiatry. But it is important to recall that neuroscience, as a science, remains a process, and in so far as people are working on the common project of explanation, the objects of knowledge need to be interpreted. Normativity cannot be expunged from science, nor should it be. *We *make sense of the world and explain it with our theories, and it is inevitable that practical considerations will play an important role in theory choice. This means that reductionism need not be the *raison d'être *for the naturalistic project, but neither should it imply that reductionism is not possible, in principle. It is important to note that defining mental content in this way becomes a practical consideration. Accordingly, behavior can be interpreted using a level of abstraction that depends upon the needs of the investigator (and/or clinician).

### Essentialism

A more controversial ontological assumption of the medical model of psychiatry is essentialism. This is the claim that psychiatric disorders, as defined by clinical nosology, map onto reality in a discrete way, and that these disorders possess essential properties, without which they would not be what they are. We argue that this assumption is highly questionable, and that as *currently conceived*, is anachronistic at best, and remains inconsistent with scientific thinking (at worst), and therefore is in need of re-examination and revision.

Science routinely organizes its body of knowledge into categories. How we sort things into categories largely depends on what measures we value. That is, we classify objects for a particular reason or to serve a specific function; to these ends, classification schemes cannot be arbitrary or random assortments. As Sadler notes, "...this non-arbitrariness is essential to a classification because it provides the basis for users with common purposes to talk about the same things. For us to discuss 'major depression' productively, we have to agree, in large part, about what major depression is, and in what practical context such a notion arises" [13].

An important concern for classification is the concept of validity. The validity of a category is related to the degree that it fits within a consonant body of explanatory theories. So, to group lungfish and cows in a similar category would require that there are genuine motivations for doing so. If one were an evolutionary biologist, such a grouping would align with what is known about macro-evolutionary processes. If one were a fisherman, the validity of such a pairing would seem impractical.

A criticism of the construct of essentialism is found in the later work of Ludwig Wittgenstein. Summarizing the Wittgensteinian view, Garth Hallett writes:

Suppose I show someone various multi-coloured pictures, and say: "The colour you see in all these is called "yellow ochre"... Then he can look *at*, point *to*, the common thing." But "compare this case: I show him samples of different shades of blue and say: "The colour that is common to all these is what I call "blue"."' Now what can be looked at or pointed to save the varied hues of blue? And don't say, "There *must *be something common, or they would not, be called 'blue,"' "but *look and see *whether there is anything in common at all" [14].

The crucial argument here is that the property of "blue" is reliant, to some extent, upon practical considerations and constraints.

Yet, a form essentialism persists in psychiatry. This is clearly articulated by Robins and Guze who claim that, "...the finding of an increased prevalence of the same disorder among the close relatives of the original patients strongly indicates that one is dealing with a valid entity" [15]. In this framework, genetic and biochemical factors are attributed as primary causes, and the role of psychiatry is to locate these pathological qualities within the physical brain. While experience does play a role in one's mental health, this model is decidedly oriented toward brain function. In this way, genetic and biochemical causes are seen as exerting their influences uni-directionally and any/all manifest symptoms are the consequence of unique and individuated etiologies.

The medical model of psychiatry views the current classifications as representing discrete organic disease states as opposed to heterogeneous symptom clusters. Validation of these symptom clusters often occurs via post-hoc quantitative and statistical analyses (such as hierarchical cluster analysis or pattern recognition paradigms) of the clinical data to ascertain which combinations of symptoms tend to group together. The problem with creating these types of discrete definitions for many contemporary psychiatric conditions is that "...no amount of clustering can get around the fact that several variables used in such models may have little or no biological plausibility" [16]. Without clear biological mechanisms, it is unclear whether symptom clusters represent different ways of labeling the same affliction, socio-cultural influences, or other biological confounds. Peter Zachar and Nick Haslam have presented a strong case that psychiatric categories do not uniformly individuate to underlying essences, but are defined, to a large part, by practical considerations [17-24]. In many ways, this recalls the Szaszian argument for mental illness as "myth" - here literally used to denote a practical, explanatory narrative.

We do not refute, or even doubt that practical considerations are important to define the threshold(s) at which a particular set of signs and symptoms may be deemed clinically relevant. But, if we are to regard essentialism as critical to the medical model of psychiatry, and adopt practice standards in accordance, then the task at hand is to establish how and what essential criteria are pertinent to any construct of normality and order (versus abnormality and disorder), as relates to brain function, mental processes and expressions of cognition, emotion and behavior (within a social milieu). Toward this end, we have posited that one such "essential" element of normality is non-linear adaptive properties within and between particular brain networks; thus progressive linearity would be aberrant and could manifest effects from the cellular to the cognitive-behavioral (and even socio-cultural) levels [25]. In this way, mental disorders would occur as a spectrum of possible effects. We maintain that particular genotypic factors predispose endo- and exophenotypes that are differentially expressed through interaction(s) with internal and external environmental influences throughout the lifespan, thereby grounding neuropsychiatric syndromes to underlying biological factors [25,26].

This acknowledges causal determinants of psychiatric disorders (at least at formal and material levels), and while accepting a form of token physicalism (i.e.- that particular mental events occur as result of some physical function(s) or dysfunction(s)), allows for appreciation of both emergence and the bio-psychosocial influence of environments. As well, the spectrum disorder concept satisfies the criteria that define the medical model (i.e.- realism, naturalism, reductionism, essentialism). In this light, a spectrum disorder can be considered to 1) involve neural substrates (i.e.- realism); 2) represent a disturbance in the natural function of the substrate(s) or system (i.e.-. naturalism); 3) be a perturbation or disruption of some underlying and/or contributory component(s) of the bio-psychosocial organism (i.e.- reductionism - in this case as token physicalism), and 4) manifest a particular "*eidos*" that defines its aberrant qualities - in this case the progressive loss of non-linear adaptability and the resultant effects on neural function, cognition, emotion and behavior (i.e.- essentialism).

## Conclusion

Psychiatry has increasingly adopted a categorical approach in delineating mental disorders. This has been beneficial insofar as the defined categories reflect clear and well-understood biological mechanisms. For certain psychiatric conditions, such as schizophrenia, bipolar disorder, and other psychoses that involve clear dysfunctions of mechanisms that regulate perception, cognition, and communication, a categorical approach may be reasonable [2]. Human beings, however, have a range of behaviors whose normality or pathology is constrained within certain socio-cultural niches. Various phobias, compulsions, obsessions, and emotions cannot easily be explained by a singular biological mechanism. As well, manifestations of the same condition may be the result of heterogeneous mechanisms working in concert.

Essentialism is evidently important to the medical model, and as such persists in contemporary psychiatry. One of the central tenets in essentialism is the existence of natural kinds. According to Zachar, a natural kind is "...an entity that is regular (nonrandom) and internally consistent from one instance to the next" [24]. That is, once the property that captures the essence of a specific natural kind is known, that property can identify any other prototypical instantiation of that kind with accuracy. But, if a category cannot be identified with respect to its essential properties, then such a category is not, in the strict definitional sense, a natural kind, but an artificial category.

Rom Harré argues that the philosophy of science is such that the idea of a 'natural kind' is a fancy, and that a 'natural kind' is a concept which can only be understood within the double framework of practice and theory [27]. The validity of a category is contingent upon how well it integrates within a diverse, multidimensional system of fact(s) and explanation(s). While the theoretical context of the kind determines, via appropriate hierarchical explanations, what properties constitute an entity's essence, it is the practical context that distinguishes accidental properties from essential ones, and we opine, perhaps more importantly, what extent of properties will be deemed relevant to regard and guide action(s).

To be sure, physiological systems function and interact nonlinearly over a wide range of spatial and temporal scales. As Goldberger notes, "...the combination of nonlinearity and non-stationarity, more the rule than the exception in the output of physiologic systems, poses a major challenge to conventional bio-statistical assessments and standard reductionist modeling stratagems" [28]. Biological systems (including the embodied brain-mind) display complex network properties, and behavioral processes are often best characterized as non-linear interactions between physiological systems and the environment [29]. The extent to which the activity of the system as a whole reflects the response(s) of its component networks will vary based upon the condition of the system and its sensitivity, and relative attractors and constraints that exist; each and all of these may be differentially expressed in certain individuals, at various points throughout the lifespan. Moreover, there is evidence to suggest that the activity and response-parameters of constituent parts and networks (i.e.- "bottom-up" effects) may be responsive to, and affected by the activity of the entire system as a whole - inclusive of psycho-social factors in which it is nested (i.e- "top-down" effects) [30].

Therefore, it remains an open question whether there are essential parameters that characterize these nonlinear dynamical patterns. We believe that the aforementioned refined eidetic conceptualization shows some promise, and in this way might provide a "missing link" between the medical model and psychiatry. Further research in neuropsychiatry will need to reassess the role of spatial and temporal scales in diseased organisms. Mental disorders, like all other dysfunctions, are *processes *that unfold through time. It is important to heed Ghaemi's advice, and recall that etiology is not a binary issue, but instead involves elements of degree [31]. In light of this, we posit that one of the benefits of the spectrum concept is that it allows categorization of mental disorders according to the extent and type(s) of relatedness conferred by 1) common genetic risk and predisposing factors, 2) dysfunction of shared substrates and networks, and 3) benefit from types of treatments that have identifiable effects/actions.

An understanding of mental normality and pathology necessitates an approach that embeds it in the complex spatial and temporal processes of life. Yet, we must be cautious - despite the attractiveness and popularity of complexity science, it is important to ground any such account to well-established fact(s), and appreciate the limits of what is known and un-known. As Jaspers noted, "every concrete event - whether of a physical or psychic nature - is open to causal explanation in principle, and psychic processes too may be subjected to such explanation. There is no limit to the discovery of causes and with every psychic event we always look for cause and effect" [32], but he also adds that "...reality is seen through the spectacles of one theory or another. We have therefore to make a continual effort to discount theoretical prejudices...and to train ourselves to pure appreciation of facts...every advance in factual knowledge means an advance in method..." [33]

At some point, the distinction between what is normal and abnormal, ordered and disordered will need to be made, and any such distinction must be practical in the sense of its viability to sustain the good of patient-centered clinical care. Therefore, it may be that the task (for the Decade of the Mind project, development of the *DSM-V*, and for psychiatry, if not medicine, writ large) is to clarify how syndromes are related (within various spectrum disorders), and adapt or create a classification scheme, nomenclature (and thus ontology) that communicates the meaning and value of taxonomy and diagnosis. Whether an attempt to elucidate the "natural basis" of mental function and dysfunction will serve such practical ends remains to be seen, and thus, this goal remains a work in progress.

## Competing interests

The authors declare that they have no competing interests.

## Authors' contributions

Both authors contributed to the writing and critical revision of the manuscript.

## About the Authors

Tejas Patil is a medical student at the University of Southern California's Keck School of Medicine, and formerly was a Research Scholar at the Center for Neurotechnology Studies, Potomac Institute for Policy Studies, Arlington, VA. He is interested in the philosophy of mind and neuroscience.

James Giordano, PhD is Editor-in-Chief of *PEHM*. He is Director of the Center for Neurotechnology Studies and Chair of Academic Programs at the Potomac Institute for Policy Studies, Arlington, VA, USA; he is a Research Associate at the Wellcome Centre for Neuroethics and Uehiro Centre for Practical Philosophy, University of Oxford, Oxford, UK.
